# Supplementary material for: Risk of Lymphedema and Death after Lymph Node Dissection with Neoadjuvant and Adjuvant Treatments in Patients with Breast Cancer: An Eight-Year Nationwide Cohort Study
Source: Healthcare (Basel). 2023 Jun 23;11(13):1833. doi: 10.3390/healthcare11131833 (PMC10340475; doi:10.3390/healthcare11131833)

Supplementary Materials

Method S1: Methodology of sensitivity analysis

We conducted seven prespecified sensitivity analyses. First, taxane-based NAC was independently included as a covariate in the Cox regression model. Second, prespecified comorbidities were included as covariates in the survival analysis. Third, a survival analysis was conducted using the unmatched cohort to examine the impact of NAR on the risk of breast cancer-related LE from all causes. Fourth, the LE risk was compared between the reoperation and SLNB groups. Fifth, the definition of the intervention was modified to a composite of surgeries within 60 days to identify patients in the reoperation group by the overall invasiveness of lymph node dissection, regardless of the number of surgeries. That is, patients who underwent both SLNB and ALND were included in the reoperation group in the main analysis but in the ALND group in the sensitivity analysis. Sixth, the time-varying regression model examined an interaction effect between the type of surgery and duration of adjuvant radiotherapy and chemotherapy. Finally, a univariate analysis was conducted on patients with outcomes on the adjuvant chemotherapy and radiotherapy duration.

Table S1: Codes used to define the inclusion/exclusion criteria, exposures, baseline covariates, time-varying covariates, and outcomes of interest.

Diagnosis	ICD-10-CM code and definition	Diagnostic definition
**Inclusion criteria**		
Breast cancer	C50	Initial diagnosis between 01/01/2011 and 12/31/2013 [entry date]
**Exclusion criteria**		
Cancer	C50	Diagnosis between 01/01/2002 and 12/31/2010
	C00, C01, C02, C03, C04, C05, C06, C07, C08, C09, C10, C11, C12, C13, C14, C15, C16, C17, C18, C19, C20, C21, C22, C23, C24, C25, C26, C30, C31, C32, C33, C34, C37, C38, C39, C40, C41, C43, C45, C46, C47, C48, C49, C51, C52, C53, C54, C55, C56, C57, C58, C60, C61, C62, C63, C64, C65, C66, C67, C68, C69, C70, C71, C72, C73, C74, C75, C76, C77, C78, C79, C80, C81, C82, C83, C84, C85, C88, C90, C91, C92, C93, C94, C95, C96, C97	Diagnosis within one year prior to diagnosis of breast cancer
Surgeries	mastectomy, lumpectomy, axillary dissections	Breast cancer-related surgery within one year prior to diagnosis of breast cancer
Lymphedema	I89.0, I97.2, CDT	Diagnosis within one year prior to diagnosis and before the index date
Death		Reports of death before the index date
**Exposure**		
Breast cancer-related surgery	SLNB group	Lumpectomy or mastectomy with SLNB in one day [index date]
	ALND group	Lumpectomy or mastectomy with ALND in one day [index date]
	Re-operation group	More than one lumpectomy or mastectomy over 60 days after the index date [index date, index date + 60]
**Baseline covariates**		

**Neoadjuvant therapies**		
Neoadjuvant radiotherapy[1]		Radiotherapies between the cohort entry date and index date
Neoadjuvant chemotherapy		Chemotherapies between the cohort entry date and index date<br>Taxanes prescribed between the cohort entry date and index date
**Comorbid conditions within one-year period before initial breast cancer diagnosis**		
Diabetes mellitus (both with and without complications)	E10, E11, E12, E13, E14	
Chronic back pain	M51, M53, M54	
Osteoarthritis	M15, M16, M17, M18, M19	
Rheumatoid	M05, M06	
Osteoporosis	M80, M81, M82	
Chronic obstructive pulmonary disease (COPD)	J43, J44	
Dementia	F00, F01, F02, F03, G30	
Schizophrenia	F20, F21	
Depressive disorders	F32, F33	
Anxiety disorders	F40, F41	
Sleep disorder	G47, F51	
Hyperlipidemia	E78	
Hypertension	I10, I11, I12, I13, I15	
Cardiovascular diseases	I05, I06, I07, I08, I09, I20, I21, I22, I23, I24, I25, I26, I27, I30, I31, I32, I33, I34, I35, I36, I37, I38, I39, I40, I41, I42, I43, I44, I45, I46, I47, I48, I49, I50, I51, I52	
Renal failure	N17, N18, N19	
Chronic liver diseases	K72, K73, K74, K75, K76, K77	
Cerebrovascular disease	I60, I61, I62, I63, I64, I65, I66, I67, I68, I69	
Anemia	D50, D51, D52, D53, D55, D56, D57, D58, D59, D60, D61, D62, D63, D64	
**Charlson Comorbidity Index (Score)**		
Myocardial infarction	I21, I22, I252	
Congestive heart failure	I099, I110, I130, I132, I255, I420, I425, I426, I427, I428, I429, I43, I50, P290	
Peripheral vascular disease	I70, I71, I731, I738, I739, I771, I790, I792, K551, K558, K559, Z958, Z959	

Cerebrovascular disease	G45, G46, I60, I61, I62, I63, I64, I65, I66, I67, I68, I69, H340	
Dementia	F00, F01, F02, F03, G30, F051, G311	
Chronic pulmonary disease	I278, I279, J40, J41, J42, J43, J44, J45, J46, J47, J60, J61, J62, J63, J64, J65, J66, J67, J684, J701, J703	
Connective tissue disease (Rheumatologic disease)	M05, M06, M315, M32, M33, M34, M351, M353, M360	
Peptic ulcer disease	K25, K26, K27, K28	
Mild liver disease	B18, K700, K701, K702, K703, K709, K713, K714, K715, K717, K73, K74, K760, K762, K763, K764, K768, K769, Z944	
Moderate or severe liver disease (3)	I850, I859, I864, I982, K704, K711, K721, K729, K765, K766, K767	
Diabetes without complications	E100, E101, E106, E108, E109, E110, E111, E116, E118, E119, E120, E121, E126, E128, E129, E130, E131, E136, E138, E139, E140, E141, E146, E148, E149	
Diabetes with complications (2)	E102, E103, E104, E105, E107, E112, E113, E114, E115, E117, E122, E123, E124, E125, E127, E132, E133, E134, E135, E137, E142, E143, E144, E145, E147	
Paraplegia and hemiplegia (2)	G041, G114, G800, G801,G802, G81, G82, G830, G831, G832, G833, G834, G839	
Renal disease (2)	I120, I131, N030, N031, N032, N033, N034, N035, N036, N037, N038, N039, N050, N051, N052, N053, N054, N055, N056, N057, N058, N059, N18, N19, N250, Z490, Z491, Z492, Z940, Z992	
Cancer (2)	C00, C01, C02, C03, C04, C05, C06, C07, C08, C09, C10, C11, C12, C13, C14, C15, C16, C17, C18, C19, C20, C21, C22, C23, C24, C25, C26, C30, C31, C32, C33, C34, C37, C38, C39, C40, C41, C43, C45, C46, C47, C48, C49, C50, C51, C52, C53, C54, C55, C56, C57, C58, C60, C61, C62, C63, C64, C65, C66, C67, C68, C69, C70, C71, C72, C73, C74, C75, C76, C81, C82, C83, C84, C85, C88, C90, C91, C92, C93, C94, C95, C96, C97	
Metastatic carcinoma (6)	C77, C78, C79, C80	
AIDS/HIV (6)	B20, B21, B22, B24	
**Demographic variables**		
Age group		<35, 35-44, 45-54, 55-64, 65-74, 75-84, ≥85
Sex		
Type of Insurance	NHIS<br>Medical Aid	Classified based on annual health premiums proportional to household incomes
**Time-varying covariates**		
**Adjuvant therapies**		
Adjuvant radiotherapy		Number of adjuvant radiotherapies during follow-up

Adjuvant chemotherapy		Number of adjuvant chemotherapies during follow-up
**Outcome**		
Lymphedema	I89.0, I97.2	Followed up for 4 years since the index date
All-cause mortality		Followed up for 4 years since the index date

[1]Excluded from the main analysis due to lack of patients after propensity score matching.
Abbreviations: ICD-10, International Classification of Diseases 10th edition; CDT, complete decongestive therapy; SLNB, sentinel lymph node biopsy; ALND, axillary lymph node dissection; NHIS, National Health Insurance Service

Table S2: Adjuvant treatments received until death or the end of the 4-year follow-up

Variables	Before propensity score matching						p-value
	SLNB (n = 6,708)		ALND (n = 32,479)		Reoperation within 60 days* (n = 604)		
	N	%	N	%	N	%	
**Adjuvant radiotherapy**[1]							<.0001
Yes	4,919	73.3	22,904	70.5	425	70.4	
**Number of sessions**							<.0001
1-20	1,567	23.36	4,687	14.43	74	12.25	
21-40	3,321	49.51	17,893	55.09	341	56.46	
41-	31	0.46	324	1	10	1.66	
**Adjuvant chemotherapy**[1]							<.0001
Yes	3,327	49.6	21,810	67.2	358	59.3	
**Number of sessions**							<.0001
1-4	1,342	20.01	4,902	15.09	79	13.08	
5-14	1,260	18.78	10,555	32.5	202	33.44	
15-	725	10.81	6,353	19.56	77	12.75	

[1]Followed up until death or the end of the four-year follow-up period.

Abbreviations: SLNB, sentinel lymph node biopsy; ALND, axillary lymph node dissection

Table S3: Incidence Rate Ratio of the patients in the subgroups

Outcome	Group	No. of events	Person-years	Incidence Rate per 100 person-years	Crude IRR	95% CI	p-value
**Lymphedema**	**SLNB**	641	24,618	2.604	0.496	(0.450 - 0.545)	<.0001
	**ALND**	1,194	22,725	5.254			
**Death**	**SLNB**	145	26,417	0.549	0.793	(0.637 - 0.986)	0.037
	**ALND**	182	26,289	0.692			

Abbreviations: IRR: Incidence rate ratio; CI: confidence interval; SLNB, sentinel lymph node biopsy; ALND, axillary lymph node dissection

Table S4: Association of prespecified comorbidities with lymphedema and death risk

Covariates	Lymphedema HR (95% CI)	Death HR (95% CI)
**Type of surgery (vs. ALND)**		
SLNB	0.506 (0.460 - 0.557)***	0.790 (0.634 - 0.984)*
**Age (vs. < 35)**		
35-44	0.734 (0.584 - 0.924)**	0.527 (0.293 - 0.948)*
45-54	0.831 (0.668 - 1.034)	0.510 (0.293 - 0.889)*
55-64	0.742 (0.585 - 0.940)*	0.839 (0.473 - 1.488)
65-74	0.611 (0.463 - 0.807)***	1.631 (0.898 - 2.963)
75-84	0.480 (0.321 - 0.717)***	3.351 (1.757 - 6.390)***
85 ≤	0.293 (0.071 - 1.214)	17.326 (8.112 - 37.006)***
**Sex**		
Male	1.136 (0.424 - 3.043)	0.920 (0.282 - 3.004)
**Types of insurance (vs. NHIS)**		
Medical Aid	1.104 (0.825 - 1.479)	2.053 (1.378 - 3.057)***
**Prespecified comorbid conditions 1 year before the onset**		
Anemia	0.886 (0.624 - 1.224)	0.450 (0.161 - 1.257)
Anxiety disorders	1.123 (0.825 - 1.529)	0.714 (0.316 - 1.614)
Cardiovascular diseases	1.491 (1.164 - 1.911)**	1.170 (0.74 - 1.848)
Cerebrovascular disease	0.844 (0.585 - 1.218)	1.018 (0.603 - 1.719)
Chronic back pain	1.106 (0.997 - 1.227)	0.804 (0.621 - 1.039)
Chronic liver diseases	1.076 (0.832 - 1.392)	1.718 (1.047 - 2.819)*
COPD	0.935 (0.444 - 1.968)	1.262 (0.451 - 3.535)
Dementia & Alzheimer	1.565 (0.688 - 3.561)	2.825 (1.458 - 5.475)**
Depressive disorders	0.984 (0.707 - 1.368)	1.041 (0.531 - 2.044)
Diabetes mellitus	1.166 (0.962 - 1.415)	1.237 (0.886 - 1.728)
Hyperlipidemia	0.823 (0.659 - 1.027)	1.056 (0.672 - 1.658)
Hypertension	1.080 (0.947 - 1.232)	0.995 (0.758 - 1.307)
Osteoarthritis	1.199 (1.050 - 1.370)**	1.088 (0.812 - 1.457)
Osteoporosis	0.938 (0.725 - 1.214)	0.680 (0.386 - 1.199)
Renal failure	0.744 (0.353 - 1.566)	5.342 (2.973 - 9.601)***
Rheumatoid	1.003 (0.675 - 1.492)	1.162 (0.478 - 2.827)
Schizophrenia	0.604 (0.195 - 1.876)	3.25 (0.806 - 13.098)
Sleep disorder	0.921 (0.666 - 1.273)	0.947 (0.508 - 1.763)

*p-value < 0.05, **p-value < 0.01, ***p-value < 0.001

Abbreviations: SLNB, sentinel lymph node biopsy; ALND, axillary lymph node dissection; HR, hazard ratio; CI, confidence interval; NHIS, National Health Insurance Service; COPD, chronic obstructive pulmonary disease

Table S5: Association of neoadjuvant taxane therapy with lymphedema and death risk

Covariates	Lymphedema HR (95% CI)	Death HR (95% CI)
Type of surgery (vs. ALND)		
SLNB	0.507 (0.460 - 0.558)***	0.781 (0.627 - 0.972)*
Age (vs. < 35)		
35-44	0.786 (0.624 - 0.989)*	0.636 (0.351 - 1.154)
45-54	0.928 (0.745 - 1.155)	0.646 (0.367 - 1.135)
55-64	0.877 (0.697 - 1.104)	1.117 (0.631 - 1.976)
65-74	0.779 (0.599 - 1.011)	2.245 (1.256 - 4.013)**
75-84	0.678 (0.463 - 0.991)*	5.156 (2.785 - 9.546)***
85 ≤	0.395 (0.097 - 1.608)	31.769 (15.793 - 63.908)***
Sex		
Male	1.197 (0.448 - 3.198)	1.110 (0.342 - 3.607)
Type of insurance (vs. NHIS)		
Medical Aid	1.121 (0.838 - 1.498)	2.175 (1.471 - 3.215)***
Neoadjuvant therapies		
Chemotherapies		
Taxanes	2.527 (1.944 - 3.286)***	6.093 (3.768 - 9.853)***

*p-value < 0.05, **p-value < 0.01, ***p-value < 0.001

Abbreviations: SLNB, sentinel lymph node biopsy; ALND, axillary lymph node dissection; HR, hazard ratio; CI, confidence interval; NHIS, National Health Insurance Service

Table S6. Multivariate Cox proportional hazard models before matching

	Associations with lymphedema during the four-year follow-up						Associations with death during the four-year follow-up					
Covariates	Model 1 (Unadjusted) HR (95% CI)	Model 2[1] HR (95% CI)	Model 3[2] HR (95% CI)	Model 4[3] HR (95% CI)	Model 5[4] HR (95% CI)	Model 6[5] HR (95% CI)	Model 1 (Unadjusted) HR (95% CI)	Model 2[1] HR (95% CI)	Model 3[2] HR (95% CI)	Model 4[3] HR (95% CI)	Model 5[4] HR (95% CI)	Model 6[5] HR (95% CI)
Type of surgery (vs. ALND)												
SLNB	0.380 (0.350 - 0.412)***	0.378 (0.349 - 0.410)***	0.378 (0.349 - 0.410)***	0.422 (0.389 - 0.458)***	0.424 (0.391 - 0.460)***	0.411 (0.379 - 0.446)***	0.593 (0.500 - 0.702)***	0.603 (0.509 - 0.714)***	0.603 (0.509 - 0.714)***	0.755 (0.636 - 0.897)**	0.768 (0.646 - 0.912)**	0.740 (0.623 - 0.879)***
Age (vs. < 35)												
35-44		0.908 (0.813 - 1.014)	0.908 (0.813 - 1.014)	0.960 (0.859 - 1.072)	0.982 (0.879 - 1.097)	0.993 (0.889 - 1.109)		0.648 (0.488 - 0.860)**	0.647 (0.487 - 0.859)**	0.724 (0.545 - 0.962)*	0.763 (0.574 - 1.014)	0.789 (0.594 - 1.049)
45-54		0.925 (0.832 - 1.029)	0.922 (0.828 - 1.025)	0.997 (0.896 - 1.109)	1.03 (0.925 - 1.146)	1.043 (0.937 - 1.161)		0.655 (0.500 - 0.858)**	0.652 (0.497 - 0.854)**	0.765 (0.583 - 1.003)	0.820 (0.624 - 1.077)	0.869 (0.662 - 1.141)
55-64		0.903 (0.808 - 1.009)	0.894 (0.799- 0.999)*	0.982 (0.878 - 1.097)	1.021 (0.912 - 1.142)	1.037 (0.927 - 1.160)		0.852 (0.645 - 1.125)	0.841 (0.636 - 1.111)	1.024 (0.774 - 1.353)	1.117 (0.843 - 1.479)	1.187 (0.897 - 1.572)
65-74		0.726 (0.640 - 0.823)***	0.712 (0.627 - 0.808)***	0.820 (0.722 - 0.930)**	0.852 (0.751 - 0.968)*	0.857 (0.755 - 0.973)*		1.487 (1.121 - 1.973)**	1.448 (1.089 - 1.926)*	1.945 (1.463 - 2.585)***	2.148 (1.612 - 2.863)***	2.253 (1.690 - 3.002)***
75-84		0.636 (0.532 - 0.762)***	0.620 (0.518 - 0.744)***	0.775 (0.647 - 0.928)**	0.800 (0.668 - 0.959)*	0.773 (0.646 - 0.927)**		3.381 (2.507 - 4.559)***	3.273 (2.419 - 4.428)***	5.101 (3.764 - 6.912)***	5.536 (4.076 - 7.518)***	5.561 (4.098 - 7.546)***
85 ≤		0.575 (0.331 - 1.001)	0.565 (0.325 - 0.982)*	0.718 (0.413 - 1.249)	0.74 (0.425 - 1.287)	0.709 (0.408 - 1.234)		11.661 (7.837 - 17.853)***	11.381 (7.637 - 16.962)***	18.965 (12.675 - 28.377)***	20.445 (13.640 - 30.647)***	20.064 (13.402 - 30.038)***
Sex												
Male		0.602 (0.362 - 0.999)*	0.599 (0.361 - 0.994)*	0.624 (0.376 - 1.036)	0.627 (0.377 - 1.041)	0.617 (0.372 - 1.025)		1.172 (0.644 - 2.134)	1.174 (0.644 - 2.139)	1.191 (0.653 - 2.169)	1.204 (0.660 - 2.194)	1.213(0.666 - 2.210)
Type of insurance (vs. NHIS)												

Medical Aid		1.066 (0.939 - 1.210)	1.056 (0.930 - 1.199)	1.037 (0.913 - 1.177)	1.055 (0.929 - 1.198)	1.070 (0.942 - 1.215)		1.982 (1.623 - 2.420)***	1.965 (1.608 - 2.400)***	1.925 (1.578 - 2.350)***	1.993 (1.632 - 2.434)***	1.967 (1.611 - 2.402)***
CCI												
1-2			1.031 (0.963 - 1.104)						0.940 (0.800 - 1.103)			
3			1.174 (1.041 - 1.323)**						1.382 (1.112 - 1.718)**			
Neoadjuvant therapies												
Radiotherapies				1.084 (0.706 - 1.665)						8.432 (5.768 - 12.327)***		
1-20					1.041 (0.610 - 1.777)						4.126 (2.477 - 7.176)***	
21-40					0.964 (0.459 - 2.026)						8.912 (4.894 - 16.229)***	
Chemotherapies				1.833 (1.746 - 1.924)***						2.976 (2.646 - 3.347)***		
1-4					1.622 (1.529 - 1.721)***						2.364 (2.045 - 2.733)***	
5-14					2.230 (2.084 - 2.385)***						4.027 (3.446 - 4.705)***	
15					1.796 (1.121 - 2.878)*						16.827 (10.601 - 26.712)***	
Taxanes						2.214 (2.085 - 2.352)***						4.67 (4.101 - 5.317)***

[1]**Model 1:** Univariate Cox regression

[2]**Model 2:** Multivariate Cox regression adjusted for demographic variables (age groups, sex, and types of health insurance)

[3]**Model 3:** Multivariate Cox regression adjusted for demographic variables (age group, sex, and type of health insurance) and CCI
[4]**Model 4:** Multivariate Cox regression adjusted for demographic variables (age group, sex, and type of health insurance), neoadjuvant radiotherapy, and neoadjuvant chemotherapy
[5]**Model 5:** Multivariate Cox regression adjusted for demographic variables (age group, sex, and type of health insurance) and number of NAC and NAC sessions
Model 6: Multivariate Cox regression adjusted for demographic variables (age group, sex, and type of health insurance) and neoadjuvant taxane treatment
*p-value < 0.05, **p-value < 0.01, ***p-value < 0.001
Abbreviations: SLNB, sentinel lymph node biopsy; ALND, axillary lymph node dissection; HR, hazard ratio; CI, confidence interval; NHIS, National Health Insurance Service; CCI, Charlson comorbidity index

Table S7: Multivariate Cox proportional hazards model comparing the SLNB and reoperation groups using cohort before matching

	Associations with lymphedema during the four-year follow-up					Associations with death during the four-year follow-up				
Covariates	Model 1 (Unadjusted)	Model 2[1]	Model 3[2]	Model 4[3]	Model 5[4]	Model 1 (Unadjusted)	Model 2[1]	Model 3[2]	Model 4[3]	Model 5[4]
	HR (95% CI)	HR (95% CI)	HR (95% CI)	HR (95% CI)	HR (95% CI)	HR (95% CI)	HR (95% CI)	HR (95% CI)	HR (95% CI)	HR (95% CI)
Type of surgery (vs. Reoperation)										
SLNB	0.312 (0.260 - 0.374)***	0.308 (0.257 - 0.370)***	0.308 (0.256 - 0.369)***	0.315 (0.262 - 0.379)***	0.329 (0.273 - 0.396)***	0.720 (0.430 - 1.205)	0.600 (0.357 - 1.009)	0.596 (0.354 - 1.002)	0.603 (0.358 - 1.015)	0.740 (0.434 - 1.262)
Age (vs. < 35)										
35-44		1.249 (0.845 - 1.848)	1.256 (0.849 - 1.858)	1.294 (0.875 - 1.915)	1.308 (0.884 - 1.936)		0.392 (0.185 - 0.833)*	0.392 (0.185 - 0.833)*	0.423 (0.199 - 0.900)*	0.455 (0.213 - 0.972)*
45-54		1.299 (0.889 - 1.900)	1.298 (0.888 - 1.899)	1.358 (0.929 - 1.987)	1.363 (0.931 - 1.994)		0.389 (0.193 - 0.782)**	0.387 (0.192 - 0.779)**	0.380 (0.187 - 0.771)**	0.452 (0.224 - 0.916)*
55-64		1.208 (0.812 - 1.795)	1.186 (0.797 - 1.765)	1.270 (0.854 - 1.890)	1.282 (0.861 - 1.908)		0.825 (0.412 - 1.652)	0.816 (0.407 - 1.636)	0.915 (0.456 - 1.837)	0.999 (0.495 - 2.019)
65-74		1.191 (0.769 - 1.843)	1.153 (0.743 - 1.788)	1.290 (0.852 - 1.999)	1.286 (0.830 - 1.995)		1.194 (0.574 - 2.484)	1.173 (0.562 - 2.449)	1.389 (0.664 - 2.905)	1.544 (0.731 - 3.261)
75-84		1.291 (0.732 - 2.277)	1.245 (0.704 - 2.201)	1.439 (0.815 - 2.541)	1.401 (0.793 - 2.474)		3.354 (1.566 - 7.181)**	3.294 (1.530 - 7.093)**	4.144 (1.915 - 8.968)***	4.398 (2.021 - 9.573)***
85 ≤		0.688 (0.093 - 5.085)	0.669 (0.09 - 4.949)	0.713 (0.096 - 5.268)	0.744 (0.101 - 5.500)		23.802 (10.034 - 56.464)***	24.025 (10.061 - 57.370)***	26.238 (10.969 - 62.759)***	30.814 (12.823 - 74.049)***
Sex										
Male		0.428 (0.060 - 3.063)	0.440 (0.061 - 3.147)	0.446 (0.062 - 3.191)	0.439 (0.061 - 3.138)		0.319 (0.042 - 2.393)	0.326 (0.043 - 2.467)	0.368 (0.049 - 2.757)	0.332 (0.044 - 2.496)
Types of insurance (vs. NHIS)										
Medical Aid		1.621 (1.113 - 2.361)*	1.572 (1.077 - 2.294)*	1.567 (1.076 - 2.282)*	1.571 (0.078 - 2.288)*		2.418 (1.423 - 4.109)**	2.329 (1.357 - 3.999)**	2.162 (1.263 - 3.699)**	2.284 (1.347 - 3.872)**
CCI										

1-2			1.036 (0.837 - 1.283)					0.898 (0.579 - 1.394)		
3			1.370 (0.955 - 1.965)					1.346 (0.740 - 2.447)		
Neoadjuvant therapies										
Radiotherapies				0.000 (0.000 - ∞)					88.775 (30.715 - 256.581)***	
Chemotherapies				2.032 (1.626 - 2.541)***					2.831 (1.794 - 4.469)***	
Taxanes					2.495 (1.802 - 3.456)***					6.558 (3.66 - 11.75)***

Model 1: Univariate Cox regression

[1]**Model 2:** Multivariate Cox regression adjusted for demographic variables (age group, sex, and type of health insurance)

[2]**Model 3:** Multivariate Cox regression adjusted for demographic variables (age group, sex, and type of health insurance) and CCI

[3]**Model 4:** Multivariate Cox regression adjusted for demographic variables (age group, sex, and type of health insurance), neoadjuvant radiotherapy, and neoadjuvant chemotherapy

[4]**Model 5:** Multivariate Cox regression adjusted for demographic variables (age group, sex, and type of health insurance) and neoadjuvant taxane treatment

*p-value < 0.05, **p-value < 0.01, ***p-value < 0.001

Abbreviations: SLNB, sentinel lymph node biopsy; ALND, axillary lymph node dissection; HR, hazard ratio; CI, confidence interval; NHIS, National Health Insurance Service; CCI, Charlson comorbidity index

Table S8: Multivariate Cox proportional hazards models derived after propensity score matching (PSM) using a composite definition of surgical dissections within 60 days

	Associations with lymphedema during the four-year follow-up					Associations with death during the four-year follow-up				
Covariates	Model 1 (Unadjusted)	Model 2[1]	Model 3[2]	Model 4[3]	Model 5[4]	Model 1 (Unadjusted)	Model 2[1]	Model 3[2]	Model 4[3]	Model 5[4]
	HR (95% CI)	HR (95% CI)	HR (95% CI)	HR (95% CI)	HR (95% CI)	HR (95% CI)	HR (95% CI)	HR (95% CI)	HR (95% CI)	HR (95% CI)
Type of surgery (vs. ALND)										
SLNB	0.381 (0.352 - 0.413)***	0.380 (0.350 - 0.411)***	0.380 (0.350 - 0.411)***	0.423 (0.391 - 0.459)***	0.412 (0.380 - 0.447)***	0.595 (0.502 - 0.704)***	0.605 (0.511 - 0.716)***	0.604 (0.511 - 0.715)	0.756 (0.637 - 0.898)**	0.741 (0.625 - 0.880)***
Age (vs. < 35)										
35-44		0.909 (0.815 - 1.014)	0.909 (0.815 - 1.013)	0.958 (0.859 - 1.069)	0.992 (0.889 - 1.107)		0.641 (0.485 - 0.848)**	0.641 (0.485 - 0.848)**	0.715 (0.540 - 0.945)*	0.778 (0.588 - 1.030)
45-54		0.922 (0.830 - 1.024)	0.919 (0.827 - 1.021)	0.991 (0.891 - 1.101)	1.037 (0.933 - 1.152)		0.644 (0.494 - 0.841)**	0.641 (0.491 - 0.837)**	0.748 (0.573 - 0.978)*	0.848 (0.648 - 1.109)
55-64		0.900 (0.806 - 1.005)	0.892 (0.799 - 0.996)*	0.976 (0.874 - 1.089)	1.032 (0.924 - 1.152)		0.858 (0.653 - 1.128)	0.848 (0.644 - 1.116)	1.026 (0.780 - 1.350)	1.187 (0.901 - 1.565)
65-74		0.727 (0.642 - 0.823)***	0.714 (0.630 - 0.810)***	0.818 (0.722 - 0.927)**	0.857 (0.756 - 0.971)*		1.466 (1.109 - 1.938)**	1.430 (1.080 - 1.895)*	1.907 (1.440 - 2.526)***	2.205 (1.661 - 2.928)***
75-84		0.646 (0.542 - 0.772)***	0.632 (0.529 - 0.755)***	0.784 (0.656 - 0.937)**	0.784 (0.656 - 0.937)**		3.327 (2.475 - 4.471)***	3.229 (2.395 - 4.354)***	4.985 (3.692 - 6.732)***	5.428 (4.014 - 7.341)***
85 ≤		0.571 (0.329 - 0.993)*	0.562 (0.323 - 0.977)*	0.709 (0.408 - 1.234)	0.702 (0.404 - 1.222)		11.588 (7.807 - 17.199)***	11.329 (7.622 - 16.839)***	18.705 (12.535 - 27.911)***	19.771 (13.243 - 29.517)***
Sex										
Male		0.588 (0.354 - 0.977)*	0.586 (0.353 - 0.973)*	0.609 (0.367 - 1.012)	0.603 (0.363 - 1.002)		1.151 (0.632 - 2.096)	1.155 (0.634 - 2.104)	1.172 (0.643 - 2.135)	1.193 (0.655 - 2.173)
Types of insurance (vs. NHIS)										

Medical Aid		1.089 (0.961 - 1.234)	1.080 (0.953 - 1.224)	1.058 (0.934 - 1.199)	1.091 (0.963 - 1.237)		1.974 (1.617 - 2.410)***	1.957 (1.603 - 2.390)***	1.913 (1.568 - 2.334)***	1.951 (1.598 - 2.382)***
CCI										
1-2			1.023 (0.955 - 1.094)					0.922 (0.795 - 1.094)		
3			1.165 (1.034 - 1.313)*					1.373 (1.105 - 1.706)**		
Neoadjuvant therapies										
Radiotherapies				1.079 (0.702 - 1.657)					8.428 (5.766 - 12.320)***	
Chemotherapies				1.831 (1.745 - 1.920)***					2.973 (2.645 - 3.341)***	
Taxanes					2.218 (2.089 - 2.354)***					4.645 (4.082 - 5.285)***

Model 1: Univariate Cox regression

[1]**Model 2:** Multivariate Cox regression adjusted for demographic variables (age groups, sex, and types of health insurance)

[2]**Model 3:** Multivariate Cox regression adjusted for demographic variables (age groups, sex, and types of health insurance) and CCI

[3]**Model 4:** Multivariate Cox regression adjusted for demographic variables (age groups, sex, and types of health insurance), neoadjuvant radiotherapy, and neoadjuvant chemotherapy

[4]**Model 5:** Multivariate Cox regression adjusted for demographic variables (age groups, sex, and types of health insurance) and neoadjuvant taxane treatments

*p-value < 0.05, **p-value < 0.01, ***p-value < 0.001

Abbreviations: SLNB, sentinel lymph node biopsy; ALND, axillary lymph node dissection; HR, hazard ratio; CI, confidence interval; NHIS, National Health Insurance Service; CCI, Charlson comorbidity index

Table S9: Time-varying regression analysis of the association of lymphedema and death with adjuvant radiotherapy during the four-year follow-up

Covariates	Lymphedema[1] HR (95% CI)	Death[2] HR (95% CI)
**Impact of one additional adjuvant radiotherapy**		
1 session for the SLNB group	1.003 (0.997 - 1.008)	0.994 (0.983 - 1.004)
1 session for the ALND group	1.002 (0.997 - 1.006)	0.994 (0.984 - 1.004)
**Risk at different numbers of sessions (vs. SLNB)**		
ALND at no radiotherapy	1.998 (1.766 - 2.260)	0.919 (0.694 - 1.216)
ALND at 10 radiotherapies	1.972 (1.790 - 2.172)	0.924 (0.740 - 1.153)
ALND at 20 radiotherapies	1.945 (1.742 - 2.173)	0.929 (0.726 - 1.188)
ALND at 30 radiotherapies	1.920 (1.645 - 2.240)	0.934 (0.667 - 1.308)
ALND at 40 radiotherapies	1.894 (1.536 - 2.336)	0.940 (0.596 - 1.482)
ALND at 50 radiotherapies	1.869 (1.427 - 2.449)	0.945 (0.526 - 1.697)
ALND at 60 radiotherapies	1.844 (1.322 - 2.572)	0.950 (0.462 - 1.954)

[1]Interaction estimate between surgeries and radiotherapy on lymphedema, $\beta = 0.001$, $p = 0.691$;
[2]Interaction estimate between surgeries and radiotherapy on death, $\beta = -0.001$, $p = 0.934$
Abbreviations: SLNB, sentinel lymph node biopsy; ALND, axillary lymph node dissection; HR, hazard ratio; CI, confidence interval

Table S10. Time-varying regression analysis of the association of lymphedema and death with adjuvant chemotherapy during the 4-year follow-up

Covariates	Lymphedema[1] HR (95% CI)	Death[2] HR (95% CI)
**Impact of one additional adjuvant chemotherapy**		
One session in the SLNB group	1.060 (1.048 - 1.073)	0.961 (0.943 - 0.980)
One session in the ALND group	1.061 (1.048 - 1.073)	0.950 (0.943 - 0.980)
**Risk at different numbers of sessions (vs. SLNB)**		
ALND at no chemotherapy	1.946 (1.740 - 2.176)	1.270 (0.946 - 1.705)
ALND at 10 chemotherapies	1.956 (1.726 - 2.217)	1.130 (0.901 - 1.417)
ALND at 20 chemotherapies	1.966 (1.550 - 2.494)	1.005 (0.684 - 1.477)
ALND at 30 chemotherapies	1.977 (1.368 - 2.856)	1.894 (0.484 - 1.652)

[1]Interaction estimate between surgeries and chemotherapy on lymphedema, $\beta = -0.001$, $p = 0.940$;
[2]Interaction estimate between surgeries and chemotherapy on death, $\beta = 0.012$, $p = 0.372$
Abbreviations: SLNB, sentinel lymph node biopsy; ALND, axillary lymph node dissection; HR, hazard ratio; CI, confidence interval

Figure S1: Kaplan–Meier curves comparing the SLNB and reoperation groups on the probability of lymphedema and survival, before matching

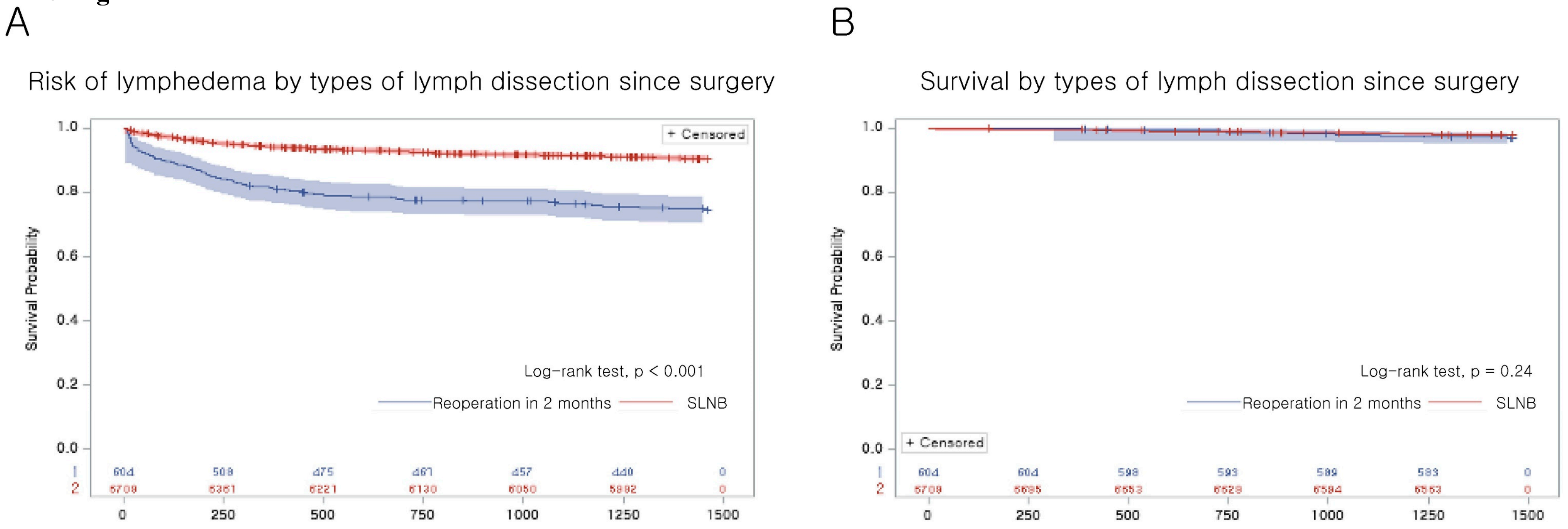

Figure S2: Univariate analysis on the subgroup of patients with either lymphedema or death vs. the number of adjuvant treatments received

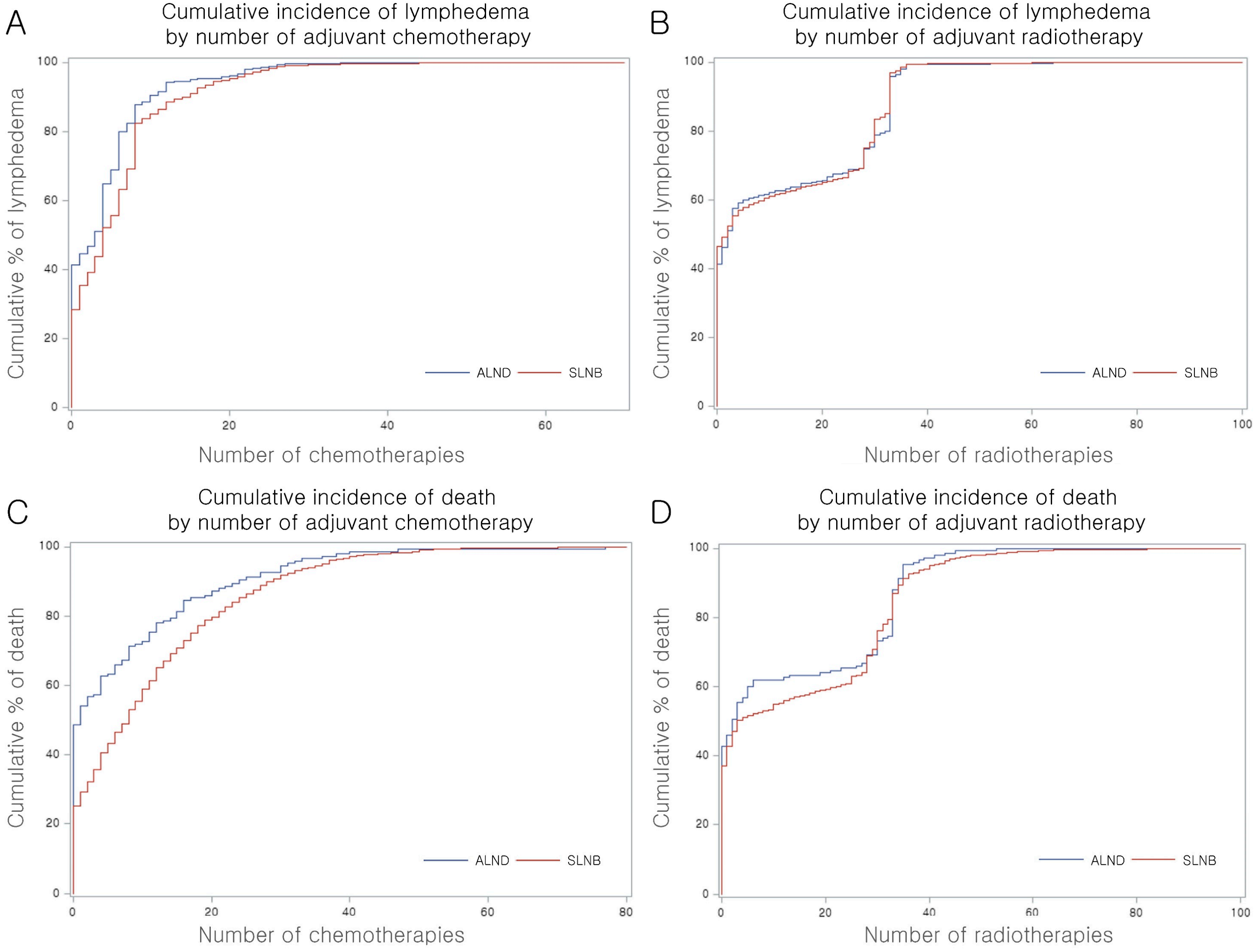

Supplement: Supplementary file 1 [file healthcare-11-01833-s001.zip › healthcare-2456132-supplementary.pdf]
